# Hyperglycemia in apolipoprotein E-deficient mouse strains with different atherosclerosis susceptibility

**DOI:** 10.1186/1475-2840-10-117

**Published:** 2011-12-28

**Authors:** Jing Li, Qian Wang, Weidong Chai, Mei-Hua Chen, Zhenqi Liu, Weibin Shi

**Affiliations:** 1 Department of Radiology and Medical Imaging, University of Virginia, Charlottesville, VA 22908, USA; 2 School of Chemical Engineering and Technology, Tianjin University, Tianjin, China, 300072; 3 Department of Medicine, University of Virginia, Charlottesville, VA 22908, USA; 4 Biochemistry & Molecular Genetics, University of Virginia, Charlottesville, VA 22908, USA

## Abstract

**Background:**

Type 2 diabetes mellitus (T2DM) is associated with an increased risk of atherosclerotic vascular disease, but it is unknown whether the other way around is true too. C57BL/6 (B6) and BALB/cJ (BALB) are two mouse strains that differ markedly in their susceptibility to atherosclerosis. In this study we investigated the development of diet-induced T2DM in these two strains.

**Methods and Results:**

When deficient in apolipoprotein E (apoE^-/-^) and fed a Western diet for 12 weeks, atherosclerosis-susceptible B6 mice developed significant hyperglycemia. In contrast, atherosclerosis-resistant BALB apoE^-/- ^mice had much lower plasma glucose levels than B6.apoE^-/- ^mice on either chow or Western diet and during an intraperitoneal glucose tolerance test. In response to glucose BALB.apoE^-/- ^mice displayed both the first and second phases of insulin secretion but the second phase of insulin secretion was absent in B6.apoE^-/- ^mice. In response to insulin B6.apoE^-/- ^mice showed a deeper and longer-lasting fall in blood glucose levels while BALB.apoE^-/- ^mice showed little reduction in glucose levels. Pancreatic islet area of BALB.apoE^-/- ^mice on light microscopy nearly doubled the area of B6.apoE^-/- ^mice. Most circulating proinflammatory cytokines were lower in BALB.apoE^-/- ^than in B6.apoE^-/- ^mice on the Western diet, as determined by protein arrays. Increased macrophage infiltration in islets was observed in B6.apoE^-/- ^mice by immunostaining for Mac2 and also by flow cytometry.

**Conclusion:**

This study demonstrates that defects in insulin secretion rather than defects in insulin resistance explain the marketed difference in susceptibility to T2DM in the B6.apoE^-/- ^and BALB.apoE^-/- ^mouse model. A smaller islet mass and more prominent islet inflammation may explain the vulnerability of B6.apoE^-/- ^mice to diet-induced diabetes.

## Background

Atherosclerosis is the principal cause of morbidity and mortality among individuals with diabetes [1,2]. The risk of coronary heart disease is two to four times higher in diabetic patients than in non-diabetic individuals [3]. Coronary heart disease, stroke, and peripheral arterial disease also occur at an earlier age in diabetic patients than in the general population [4]. Diabetic patients often have dyslipidemia, which is a key factor in the development of atherosclerosis [5], and exhibit an atherogenic lipid profile, including an enrichment of triglycerides in the HDL core [6] and an increase in small-dense, triglyceride-rich LDL particles [7].

Recent studies suggest that dyslipidemia, which comprises elevated triglyceride and LDL cholesterol levels and reduced HDL cholesterol levels, may contribute to the pathogenesis of T2DM. Supporting evidence includes observations that dyslipidemia often precedes T2DM for years, suggesting that disturbances in lipoproteins may initiate the pathological process leading to diabetes [8,9]. Individuals with low HDL have an increased risk of developing T2DM [8]. On the other hand, aerobically trained individuals have high HDL and display enhanced glucose tolerance [10]. Bezafibrate, which raises HDL levels by 16% and decreases triglyceride levels by 24%, delays the onset of T2DM and reduces the incidence of T2DM in patients with coronary artery disease [11].

One commonly used mouse model of dyslipidemia is the apolipoprotein E-deficient (apoE^-/-^) mouse, which exhibits the typical features of dyslipidemia seen in humans, including elevations in LDL cholesterol and triglyceride levels and reductions in HDL cholesterol levels [12,13]. Moreover, apoE^-/- ^mice develop all phases of atherosclerotic lesions, progressing from the early foam cell stage to the advanced stage with a fibrous cap and necrotic lipid core [14]. Recently, we have found that apoE^-/- ^mice on the C57BL/6 (B6) genetic background develop significant hyperglycemia when fed a Western-type diet [15]. As atherosclerotic cardiovascular disease is the leading cause of morbidity and mortality among patients with diabetes, apoE^-/- ^mice are obviously a more suitable model for studying diabetes than mice that do not develop atherosclerosis.

We and others have reported that genetic backgrounds have a dramatic influence on dyslipidemia [12,16,17]. BALB.apoE^-/- ^mice have much higher HDL cholesterol levels than B6.apoE^-/- ^mice, especially when fed a high fat diet, although their non-HDL cholesterol and triglyceride levels are comparable [16]. BALB.apoE^-/- ^mice also have lower levels of circulating VCAM-1 and P-selectin than B6.apoE^-/- ^mice on either chow or high fat diet [16]. Thus, we reasoned that BALB.apoE^-/- ^mice would be less susceptible to diabetes than B6.apoE^-/- ^mice due to their higher HDL and low inflammation. To test this hypothesis, in the present study we examined the development of diet-induced T2DM in the two strains as well as potential connections with inflammation.

## Methods

### Mice

Female B6.apoE^-/- ^mice were purchased from the Jackson Laboratory. Female BALB.apoE^-/- ^mice at the N12 generation were generated in our laboratory using the classical congenic breeding strategy [18]. At 6 weeks of age, mice continued with a chow diet for additional 6 weeks, or were switched onto a Western diet containing 21% fat, 0.15% cholesterol, 34.1% sucrose, 19.5% casein, and 15% starch (TD88137, Harlan Laboratories) and maintained on the diet for 12 weeks. The mice were housed in a 12 h light-12 h dark cycle pathogen-free facility at the University of Virginia. All procedures were carried out in accordance with current National Institutes of Health guidelines and approved by the University of Virginia Animal Care and Use Committee.

### Measurements of plasma glucose and insulin

Mice were fasted overnight before blood was collected through retro-orbital venous plexus puncture with the animals under isoflurane anesthesia. Plasma glucose was measured with a Sigma glucose (HK) assay kit, and insulin was measured with an ultra-sensitive ELISA kit from Crystal Chem INC.

### Glucose tolerance test (GTT) and insulin tolerance test (ITT)

GTT was performed as described by McDuffie et al [19]. Briefly, overnight fasted mice were subject to an intraperitoneal injection of 1 g glucose/kg body weight. Blood glucose levels were measured with an UltraTouch glucometer using the whole blood taken from cut tail tips immediately before and at 10, 20, 30, 60, 90, and 120 min after the injection of glucose. ITT was performed on non-fasted mice by an intraperitoneal injection of 0.75 U insulin/kg body weight. Blood glucose was also monitored as above immediately before and at 15, 30, 45, and 60 min after insulin injection.

### Glucose-stimulated insulin secretion

After being fasted overnight, mice were injected intraperitoneally with glucose (1 g/kg). Blood samples were collected from cut tail tips immediately before and at 15, 30, 60, and 120 min after the injection of glucose. Insulin concentrations in the blood were measured with the insulin kit from Crystal Chem INC.

### Plasma cytokine assay

Plasma cytokine levels of mice were analyzed using mouse cytokine arrays, which contain 144 different anti-cytokine antibodies and positive and negative controls (RayBiotech). Four separate plasma samples were pooled in an equal amount from 3 or 4 individual mice of each group, and each sample was incubated with one set of arrays. The assays were performed according to the manufacturer's instructions. The intensity of each spot on the arrays was quantified with a densitometer.

### Histological and immunohistochemical analyses

Mouse pancreas was harvested and processed as previously reported [20]. Briefly, the pancreas was fixed in 4% paraformaldehyde for > 24 h, then infiltrated overnight in 30% sucrose, and embedded in Tissue-Tech OCT compound. Cryosections (8-μm thick) were collected every 3 sections throughout the pancreas, and 4 sections were mounted on one slide. Every 5 slides were stained with haematoxylin and eosin (H&E), and the surface areas of all islets on one section per slide were counted for each mouse. The presence of macrophages in the islets was detected with a rat monoclonal antibody against mouse macrophages (MOMA-2) from Serotec, as we previously reported [21,22].

### Pancreatic islet isolation and flow cytometry analysis

Pancreatic islets were prepared using the method of Li et al [23] with a minor modification. Briefly, the pancreas was perfused in situ, first with PBS through the heart and then with a solution containing collagenase XI (1,000 U/ml) through the common bile duct. Then the entire pancreas was isolated and further incubated in the collagenase XI-containing solution at 37°C for ~15 min. Digested pancreas was filtered through a 70-μm nylon cell strainer. The captured islets were further digested into individual cells with a mixture of 500 U/ml collagenase XI and 500 U/ml collagenase I in the Hanks' salt solution at 37°C for 15~20 min. As the number of cells yielded from an individual pancreas was limited, islet cells were pooled from preparations of 2 or 3 mice.

Islet cells were incubated for 30 min on ice with the following fluorophore-conjugated primary antibodies (eBioscience, Inc): FITC-conjugated anti-CD4, pacific blue (PB)-conjugated CD8α, phycoerythrin (PE)-conjugated anti-Mac-3, allophycocyanin (APC)-conjugated anti-CD11b, and pre-captopril (preCP)-conjugated anti-CD45. Stained cells were fixed in 2% paraformaldehyde and then analyzed on a FACSCalibur flow cytometry using FlowJo software. The macrophage content was defined as cells double positive for CD11b and Mac3 on a CD45-positive gate. Lymphocytes were identified as cells positive for CD4 or CD8 on a CD45-positive gate.

### Statistical analysis

Values were expressed as means ± SE, with *n *indicating the number of animals. AVOVA or Student's t test were used for determining statistical significance between groups. Differences were considered statistically significant at *P *< 0.05.

## Results

**Figure 1:**
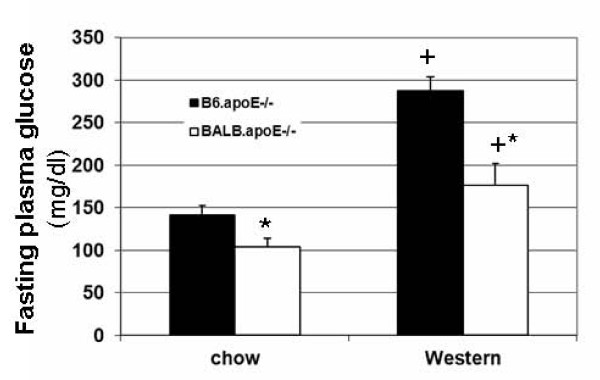
**Fasting plasma glucose levels of female B6.apoE^-/- ^and BALB.apoE^-/- ^mice when fed a chow or Western diet**. Results are means ± SE of 5 and 7 mice. Mice continued with a chow diet, or were switched onto a Western diet at 6 weeks of age and maintained on the diet for 12 weeks. * *P *< 0.05 vs. B6 mice, and ^+ ^*P *< 0.05 vs. chow diet.

Fasting plasma glucose levels of female B6.apoE^-/- ^and BALB.apoE^-/- ^mice were measured when mice were fed a chow or Western diet. As shown in Figure 1, BALB.apoE^-/- ^mice had significantly lower levels of fasting plasma glucose than B6.apoE^-/- ^mice on both chow (103.6 ± 10.9 vs. 142.0 ± 10.7 mg/dl; *P *= 0.031) and Western diets (176.9 ± 25.6 vs. 287.2 ± 17.2 mg/dl; *P *= 0.006). Compared to the chow diet, the Western diet led to significant elevations in fasting glucose levels of both B6.apoE^-/- ^(*P *= 0.00016) and BALB.apoE^-/- ^mice (*P *= 0.034).

**Figure 2:**
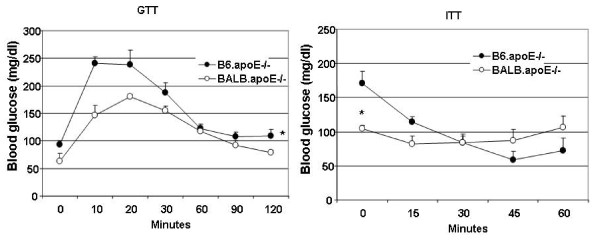
**Glucose tolerance test (GTT) and insulin tolerance test (ITT) for B6.apoE^-/- ^and BALB.apoE^-/- ^mice fed a Western diet**. For GTT, overnight fasted mice were subject to an intraperitoneal injection of glucose (1 g/kg). ITT was performed by an intraperitoneal injection of insulin (0.75 U/kg) to non-fasted mice. Blood glucose concentrations were determined with a glucometer using blood taken from cut tail tips at the indicated time points. Values are means ± SE of 4 or 5 mice. * *P *< 0.05 vs. B6 mice.

Glucose tolerance test (GTT) and insulin tolerance test (ITT) were performed on mice fed the Western diet. As shown in Figure 2, blood glucose levels over the entire time course of GTT were lower in BALB.apoE^-/- ^mice than in B6.apoE^-/- ^mice (*P *= 0.000002). Both the rise and the fall of blood glucose levels were slower in BALB.apoE^-/- ^than in B6.apoE^-/- ^mice. In response to insulin, B6.apoE^-/- ^mice showed a deeper and longer-lasting fall in blood glucose levels while BALB.apoE^-/- ^mice displayed little reduction in glucose levels. In addition, the baseline level of non-fasting blood glucose (at 0 min on ITT) was significantly lower in BALB.apoE^-/- ^than in B6.apoE^-/- ^mice (104.8 ± 4.2 vs. 170.5 ± 18.0 mg/dl; *P *= 0.038).

**Figure 3:**
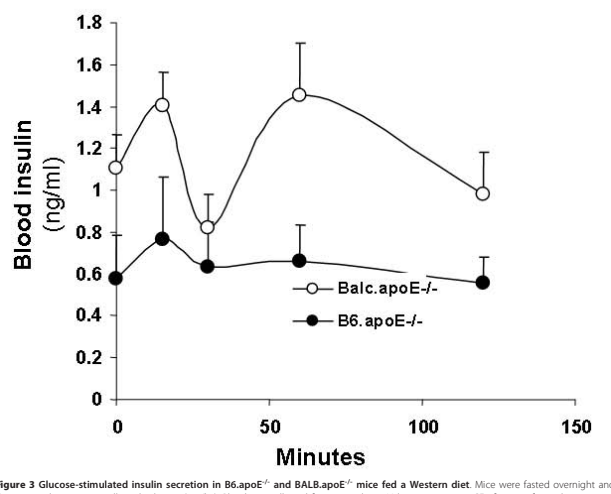
**Glucose-stimulated insulin secretion in B6.apoE^-/- ^and BALB.apoE^-/- ^mice fed a Western diet**. Mice were fasted overnight and then injected intraperitoneally with glucose (1 g/kg). Blood was collected from cut tail tips. Values are means ± SE of 5 mice for each strain.

Glucose-stimulated insulin secretion was determined on B6.apoE^-/- ^and BALB.apoE^-/- ^mice fed the Western diet. Overnight fasted mice were subject to an intraperitoneal injection of glucose (1 g/kg), and blood samples were collected from cut tail tips. Following glucose injection, BALB.apoE^-/- ^mice displayed both the first phase and the second phase of insulin secretion. In contrast, the second phase of insulin secretion was not evident in B6.apoE^-/- ^mice (Figure 3). Also, at all the time points assessed, blood insulin concentrations were higher in BALB.apoE^-/- ^mice, and the differences were statistically significant (*P *= 0.0013).

**Figure 4:**
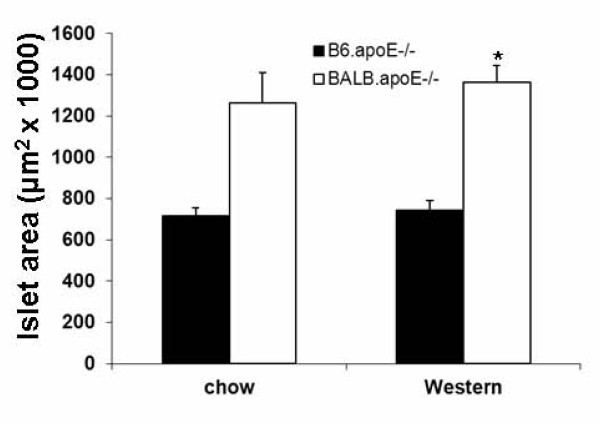
**Pancreatic islet surface areas of B6.apoE^-/- ^and BALB.apoE^-/- ^mice fed a chow or Western diet**. Cryosections of the pancreas were stained with haematoxylin and eosin. The surface area of all pancreatic islets from sections spaced approximately at 500 μm was measured for each mouse. Values are means ± SE of 3 to 10 mice. * *P *< 0.05 vs. B6 mice.

The total surface areas of islets on H&E-stained sections intervaled approximately at 500 μm across the entire pancreas were measured. BALB.apoE^-/- ^mice had a significantly larger islet surface area than B6.apoE^-/- ^mice on the Western diet (1,361,876 ± 81,840 vs. 742,591 ± 47,578 μm^2^; *P *= 0.0013) and had a trend toward larger islet surface area than B6.apoE-/- mice on the chow diet (1,264,554 ± 144,838 vs. 716,558 ± 40,582 μm^2^; *P *= 0.068) (Figure 4). The Western diet feeding had no influence on islet surface area in either strain (*P *> 0.5).

**Figure 5:**
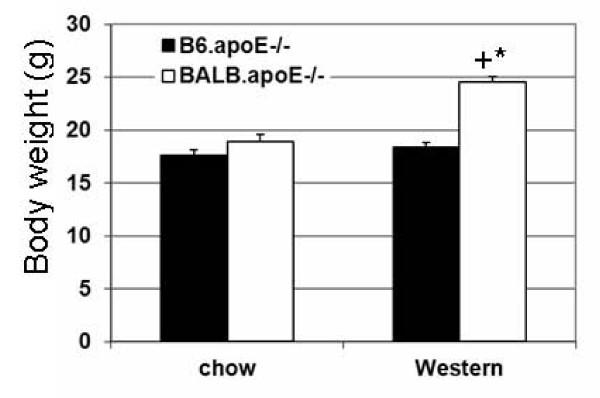
**Body weight (g) of B6.apoE^-/- ^and BALB.apoE^-/- ^mice on chow or a Western diet**. Results are means ± SE of 9 to 17 mice. * *P *< 0.05 vs. B6.apoE^-/-^, + *P *< 0.05 vs. chow diet.

At 12 weeks of age on the chow diet, BALB.apoE^-/- ^mice had a body weight comparable to B6.apoE^-/- ^mice (18.9 ± 0.7 vs. 17.6 ± 0.9 g; *P *= 0.30) (Figure 5). However, after being fed the Western diet for 12 weeks, BALB.apoE^-/- ^mice had a significantly larger body weight than B6.apoE^-/- ^mice (24.5 ± 0.5 vs. 18.4 ± 0.5 g; *P *< 0.0001). The body weight of BALB.apoE^-/- ^mice fed the Western diet was significantly larger compared to those fed the chow diet (*P *< 0.0001). By contrast, the body weight of B6.apoE^-/- ^mice did not differ statistically between the two different diets.

**Figure 6:**
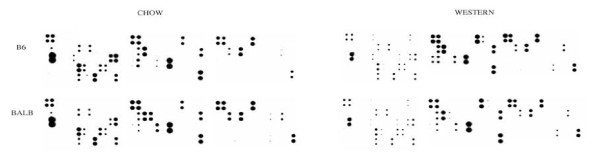
**Antibody array analysis of plasma cytokine levels in B6.apoE^-/- ^and BALB.apoE^-/- ^mice on both chow and Western diets**. Plasma samples were pooled in an equal amount from 3 mice for each group. The potion of each cytokine on the membranes corresponds to its position as shown in the supplementary table. There are both positive and negative controls on each membrane.

Plasma cytokine levels in B6.apoE^-/- ^and BALB.apoE^-/- ^mice were evaluated using RayBio mouse cytokine antibody arrays (Figure 6, Additional File 1, Table S1). On the chow diet, B6.apoE^-/- ^mice had higher levels of IGFBP-6, IL-12, LIX, L-selectin, MIP-2, P-selectin, sTNF RI, IGFBP-2, MMP-3 and osteoponin than BALB.apoE^-/- ^mice, and BALB.apoE^-/- ^mice had higher levels of IGF-I, resistin, decorin, galectin, pentraxin 3, and TWEAK R than B6.apoE^-/- ^mice. On the Western diet, B6.apoE^-/- ^mice displayed higher plasma levels of AXL, CXCL16, eotaxin-2, IL-12, TCA-3, sTNF RI, E-selectin, FcG RIIB, ICAM-1, IGFBP-2, MMP-3, osteoponin, resistin, ACE/CD143, E-cadherin, galectin, HAI-1, and pentraxin 3 than BALB.apoE^-/- ^mice, while BALB.apoE^-/- ^mice only displayed higher levels of MIP-2, IGF-I, and TWEAK R than B6.apoE^-/- ^mice.

**Figure 7:**
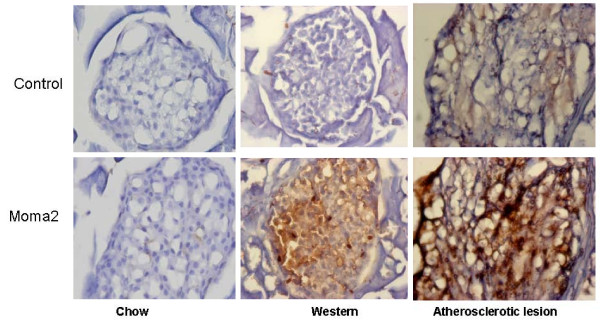
**Immunohistochemical analysis of macrophages in the islets of B6.apoE^-/- ^mice fed a chow or Western diet**. Sections were stained with a rat anti-mouse macrophage antibody for MOMA-2. Mouse aorta with atherosclerotic lesions serves as controls. The top row is negative controls. Original magnification × 40.

**Figure 8:**
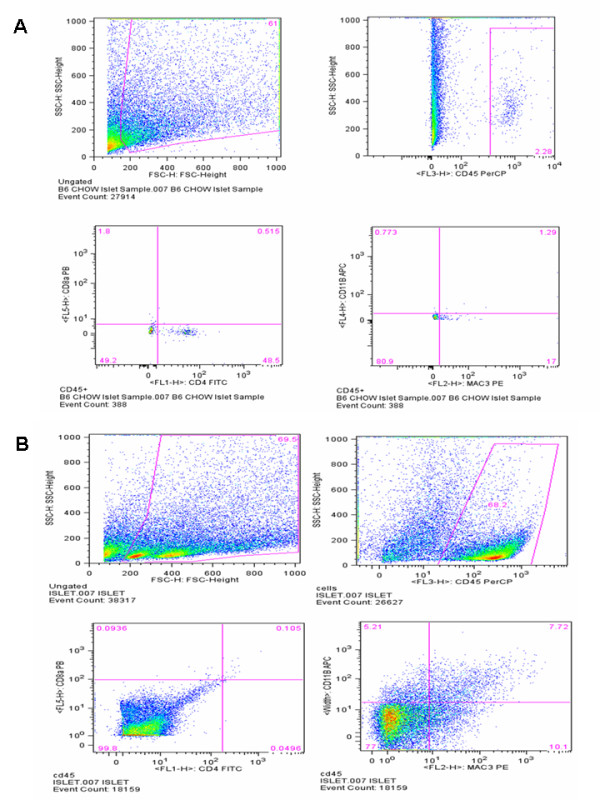
**Flow cytometric analysis of macrophages and lymphocytes in the islets of B6.apoE^-/- ^mice fed a chow (Figure 8A) or Western diet (Figure 8B)**. Macrophages were identified as CD11b and Mac3 double positive cells gated for CD45. Lymphocytes were identified as CD4 or CD8 positive cells gated for CD45. Islet cells were stained with anti-CD45, -CD4, -CD8a, -CD11b, and Mac3 antibodies.

Immunohistochemical analysis with an antibody against MOMA2 showed that the islets of B6.apoE^-/- ^mice fed a chow diet had no macrophages (Figure 7). However, in the pancreas of B6.apoE^-/- ^mice fed the Western diet, macrophages were observed within and around the islets. To accurately quantitate relative macrophage abundance, we analyzed the proportion of macrophages and lymphocytes in isolated islet cells by flow cytometry. Macrophages were identified as CD11b and Mac3 double positive cells. On the chow diet, only 1.29% living islet cells were macrophages, while on the Western diet macrophages accounted for 7.72% of the total living islet cells (Figure 8A, B). Few lymphocytes were detected in the islets of B6.apoE^-/- ^mice fed either a chow or Western diet.

## Discussion

Although T2DM is a well recognized risk factor for atherosclerotic vascular disease, it is unclear whether individuals who are susceptible to atherosclerosis would have an increased tendency to develop T2DM. We have now examined this using two mouse strains that differ markedly in susceptibility to atherosclerosis. Clear differences have been observed between the two apoE^-/- ^mouse strains in blood glucose levels under both fasting and non-fasting conditions and during the glucose tolerance test. Atherosclerosis-susceptible B6.apoE^-/- ^mice developed significant hyperglycemia on the high fat diet, whereas atherosclerosis-resistant BALB.apoE^-/- ^mice did not.

B6 and BALB are two inbred mouse strains that have been studied as a genetic model of atherosclerosis. When fed an atherogenic diet containing high fat, high cholesterol, and cholate, B6 mice readily develop fatty streak lesions in the aortic root, whereas BALB mice are highly resistant to lesion formation [24,25]. However, the cholate-containing high-fat diet induces a chronic inflammatory state, with the expression of inflammatory and oxidative stress genes in the liver [26,27], gallbladder [28], and probably other organs or tissues. To avoid the influence of the cholate diet, we constructed BALB.apoE^-/- ^mice that developed spontaneous hyperlipidemia and atherosclerosis on a low fat chow diet [16]. Compared to B6.apoE^-/- ^mice, BALB.apoE^-/- ^mice are highly resistant to atherosclerosis, developing much smaller lesions. The two apoE^-/- ^strains have comparable non-HDL cholesterol and triglyceride levels, but HDL cholesterol levels are 2-fold higher in BALB.apoE^-/- ^mice on the chow diet and are 15-fold higher on the Western diet [16]. The high HDL level should contribute, at least partially, to the resistance of BALB.apoE^-/- ^mice to diet-induced hyperglycemia.

In the present study, we found that B6.apoE^-/- ^mice but not BALB.apoE^-/- ^mice had defects in insulin secretion on the high fat diet. Insulin secretion in response to glucose is usually biphasic, consisting of a transient initial peak of insulin release, followed by a prolonged second phase. The first phase insulin response was similar in both strains, but the second phase insulin secretion was observed in BALB.apoE^-/- ^mice but not in B6.apoE^-/- ^mice. Impaired insulin secretion has also been observed in wild-type B6 mice when fed a high fat diet [29-32]. Studies of isolated islets show that insulin release defects in response to glucose are associated with impairments in K_ATP _channels and intracellular calcium flux [31]. However, as B6.apoE^-/- ^mice become diabetic only when fed the high fat diet, the diet-induced inflammation probably have exacerbated the insulin secretion defect. The feeding of a high fat diet induces a chronic inflammatory state with expression of proinflammatory genes and infiltration of macrophages in local tissues [33]. In this study we have found that B6.apoE^-/- ^mice developed more significant inflammation on the Western diet than BALB.apoE^-/- ^mice, as most differentially expressed proinflammatory cytokines were more highly expressed in B6 compared to BALB mice. In a previous study, we have also found that B6.apoE^-/- ^mice have higher levels of circulating adhesion molecules than BALB.apoE^-/- ^mice on the Western diet [16].

Adhesion molecules and chemokines are associated with the recruitment of inflammatory cells, a greater expression of these molecules is expected to lead to more leukocytes recruited to local tissues. Indeed, we have observed an increased infiltration of macrophages in the islets of B6.apoE^-/- ^mice when fed the high fat diet. The increased macrophage infiltration in islets has also been observed in wild-type B6 mice fed a high-fat diet [34]. As local inflammation damages β cells and reduces insulin secretion [35], pancreatic islet inflammation developed on the high fat diet must have contributed to pancreatic β cell functional failure observed in B6.apoE^-/- ^mice.

We found that B6.apoE^-/- ^mice had an islet mass that was approximately half the size of BALB.apoE^-/- ^mice. This small islet mass partially explains the susceptibility of B6.apoE^-/- ^mice to diet-induced diabetes. The total islet mass was comparable between mice on the chow diet and those on the Western diet for both strains. This finding is consistent with the conclusion that genetic backgrounds determine the size of the endocrine pancreas in mouse strains [36].

Despite the fact that BALB.apoE^-/- ^mice displayed significant insulin resistance on the Western diet, as evidenced by the lack of insulin response during the insulin tolerance test, they did not develop hyperglycemia. As these mice had a higher plasma insulin level, the normoglycemia appeared to be maintained by a compensatory increase in insulin output. BALB.apoE^-/- ^mice had a larger islet mass, nearly double the size of B6.apoE^-/- ^mice, thus they should have a greater secretory capacity to compensate for the insulin resistance. This observation suggests that insulin resistance alone is not sufficient for the development of hyperglycemia in the apoE^-/- ^model. On the other hand, B6.apoE^-/- ^mice did not have insulin resistance, as indicated on the insulin tolerance test, but they developed hyperglycemia, suggesting that a defect in insulin secretion is essential for the development of hyperglycemia in this model.

In this study, we found that BALB.apoE^-/- ^mice but not B6.apoE^-/- ^mice exhibited an increase in body weight on the Western diet. The increased body weight might contribute to the resistance of BALB.apoE^-/- ^mice to insulin. Previous studies have shown that apoE deficiency prevents the development of obesity in B6 mice and genetically obese Ay mice on a high fat diet [37,38]. As BALB.apoE^-/- ^mice, which are deficient in apoE, exhibited an increase in body weight, other mechanisms contributing to obesity may override the protective effect of apoE deficiency observed in our experiments.

In summary, we have demonstrated that genetic backgrounds have a dramatic influence on susceptibility to diet-induced T2DM in hyperlipidemic apoE^-/- ^mice. Defects in insulin secretion but not insulin resistance explain the susceptibility of B6.apoE^-/- ^mice to diet-induced T2DM. The relatively small pancreatic islet mass and more severe inflammation probably explain their accelerated islet β-cell functional failure on the high fat diet. The recent genome-wide association studies (GWAS) have identified new loci that are implicated in β-cell development and function, highlighting insulin secretion in the development of T2DM in humans as well [39]. Pathological studies have revealed common features, including islet inflammation and β-cell destruction, in both type 1 and type 2 diabetes [34,40].

### Limitations of the present study

Although this model can explain the high prevalence of diabetes in atherosclerotic patients, we have not demonstrated the direct link between atherosclerosis and diabetes in the apoE^-/- ^mice. In a recent study of IGF-II/LDLR^-/-^ApoB^100/100 ^mice, Heinonen et al [41] have found that diabetes has little impact on lesion size when mice developed severe atherosclerosis in the aorta and the coronary artery. As non-diabetic LDLR^-/-^ApoB^100/100 ^control mice also developed severe atherosclerosis, there was little room left to demonstrate a further lesion increase associated with the effect of diabetes. There was a room when mice develop early or medial stage of atherosclerosis, but they were not studied. Our data also do not prove that the current observation is applicable to the wild-type of mice as they were not studied. Nevertheless, a previous study has shown that most wild-type mouse strains, including B6 and BALB, do not develop significant hyperlipidemia or hyperglycemia on a high fat diet, although they exhibit increased insulin release [42].

## Competing interests

The authors declare that they have no competing interests.

## Authors' contributions

JL, ZL, and WS designed research; JL, QW, WC, MHC, and WS performed research; ZL and WS revised the manuscript critically for intellectual content; JL, QW and WS analyzed data; ZL and WS wrote the paper. All authors have read and approved submission of the final manuscript.

## Supplementary Material

Additional file 1 **Plasma cytokine levels of C57BL/6 (B6) and BALB/cJ (BALB) apoE^-/- ^mice fed a chow or Western diet as detected by cytokine arrays**. Plasma cytokine levels of B6 and BALB apoE^-/- ^mice were analyzed using mouse cytokine arrays from RayBiotech, which contain 144 different anti-cytokine antibodies and positive and negative controls. Four separate plasma samples were prepared with each sample pooled in an equal amount from 3 or 4 individual mice for each group, and each sample was incubated with one set of arrays. The assays were performed according to the manufacturer's instructions. The intensity of each spot on the arrays was quantified with a densitometer. Click here for file
